# Association Between Liver Dysfunction Markers and Mortality in Heart Failure: A Systematic Review and Meta-Analysis

**DOI:** 10.7759/cureus.106412

**Published:** 2026-04-03

**Authors:** Ali Salman, Ali Irshad, Hamza I Chughtai, Mark Atkinson, Muhammad Arslan Riaz

**Affiliations:** 1 Department of Internal Medicine, Warrington Hospital, Warrington, GBR; 2 Department of Geriatric Medicine, Warrington Hospital, Warrington, GBR; 3 Department of General Medicine, Warrington Hospital, Warrington, GBR; 4 Department of Cardiology, Punjab Institute of Cardiology, Lahore, PAK

**Keywords:** all-cause mortality, heart failure, liver dysfunction, meld score, meld-xi

## Abstract

Heart failure (HF) is a major cause of morbidity and mortality worldwide and is frequently associated with dysfunction of other organs, including the liver. Hepatic congestion and impaired perfusion from cardiac dysfunction may lead to liver injury, described as cardiohepatic syndrome. Studies suggest that liver dysfunction markers, such as the Model for End-Stage Liver Disease (MELD) and MELD excluding international normalized ratio (MELD-XI) scores, may serve as prognostic indicators in HF patients. This systematic review and meta-analysis evaluated the relationship between liver dysfunction markers and mortality outcomes in HF patients. A systematic literature search was conducted. Studies evaluating the association between liver dysfunction markers (MELD or MELD-XI scores) and mortality outcomes in adult HF patients were included. Hazard ratios (HRs) with 95% confidence intervals (CIs) were pooled using the generic inverse variance method with a random-effects model in Review Manager (RevMan) version 5.4 (The Cochrane Collaboration, Copenhagen, Denmark). Subgroup analyses were performed by liver dysfunction marker type and HF phenotype. The primary outcome was all-cause mortality. Nine studies comprising over 32,000 HF patients were included. Elevated liver dysfunction markers were significantly associated with increased mortality risk (pooled HR = 1.12, 95% CI 1.06-1.18). Substantial heterogeneity was observed (I² = 91%). Subgroup analysis showed significant associations for both MELD-XI (HR = 1.09, 95% CI 1.04-1.14) and MELD scores (HR = 1.10, 95% CI 1.06-1.14). Analysis by HF type revealed significant associations in acute HF (HR = 1.07), chronic HF (HR = 1.23), and advanced HF (HR = 1.05). Sensitivity analyses confirmed the findings, with no substantial publication bias observed. Elevated liver dysfunction markers significantly correlate with increased mortality in HF patients. Both MELD and MELD-XI scores provide prognostic information across HF populations. These findings emphasize the importance of considering hepatic dysfunction in HF assessment and suggest liver dysfunction markers may aid risk stratification. Further prospective studies are needed to determine the role of these markers in clinical decision-making.

## Introduction and background

Heart failure (HF) represents a major global health challenge and continues to be a leading cause of morbidity and mortality worldwide. Current estimates suggest that more than 64 million individuals are affected by HF globally [1], placing a substantial burden on healthcare systems due to frequent hospitalizations, progressive functional impairment, and high long-term mortality rates [2]. Despite improvements in pharmacologic therapy, device-based interventions, and multidisciplinary management strategies, the prognosis for many patients with HF remains poor, particularly in advanced stages of the disease [3]. As a result, considerable research has focused on identifying reliable prognostic markers to assist clinicians in predicting outcomes and guiding treatment strategies.

HF is a complex systemic condition that affects multiple organs beyond the cardiovascular system. Among these, the liver is particularly vulnerable to the hemodynamic and metabolic disturbances associated with HF. The interaction between cardiac dysfunction and hepatic injury is often described as the cardiohepatic syndrome, a pathophysiological process characterized by hepatic congestion, reduced hepatic perfusion, and systemic inflammation [4,5]. In patients with HF, elevated central venous pressure can lead to chronic hepatic congestion [6], whereas reduced cardiac output may impair hepatic oxygen delivery [7]. These mechanisms contribute to hepatocellular injury, cholestasis, and progressive deterioration of liver function. As a result, biochemical markers of hepatic dysfunction are frequently observed in patients with moderate to severe HF and may reflect the severity of underlying cardiovascular impairment [6,8].

Over the past decade, several liver function-based scoring systems have been investigated as potential prognostic indicators in HF. Among these, the Model for End-Stage Liver Disease (MELD) score [9] and its modified form, the MELD excluding international normalized ratio (MELD-XI) score [10], have gained particular attention. Originally developed to predict survival in patients with advanced liver disease, these scoring systems incorporate laboratory parameters such as serum bilirubin and creatinine to estimate disease severity [11]. In cardiovascular populations, MELD and MELD-XI scores have been increasingly studied as markers of multisystem organ dysfunction, reflecting the interplay between hepatic and renal impairment in HF [12]. Given that MELD includes the international normalized ratio (INR), which may be influenced by anticoagulation therapy.

In contrast, MELD-XI excludes INR; these scoring systems may differ in their clinical applicability in HF populations. Therefore, evaluating both scoring systems is important to determine their comparative prognostic utility and to assess whether one provides a more consistent or clinically applicable risk stratification tool across diverse HF populations. Several observational studies have suggested that elevated MELD or MELD-XI scores are associated with worse clinical outcomes, including increased mortality, higher rates of hospitalization, and poorer overall prognosis in patients with HF [13,14].

Despite growing interest in these markers, the evidence regarding their prognostic significance remains heterogeneous. Individual studies have reported varying magnitudes of association between liver dysfunction markers and mortality outcomes in HF populations. Differences in patient characteristics, HF phenotypes [15] (such as acute versus chronic HF or HF with preserved ejection fraction (HFpEF)), follow-up durations [16], and study methodologies may contribute to these inconsistencies. Furthermore, some studies have examined MELD scores [17], whereas others have focused on MELD-XI or related hepatic biomarkers [18,19], making it difficult to directly compare findings across the literature. These variations underscore the need for a comprehensive synthesis of the available evidence to better understand the prognostic value of hepatic dysfunction in HF.

A recent meta-analysis by Dastjerdi et al. [16] evaluated the prognostic value of liver biomarkers in patients with HFpEF. However, that study was limited to a specific HF subtype and primarily focused on conventional liver biomarkers such as albumin and transaminases. In contrast, the present meta-analysis includes a broader spectrum of HF populations (acute, chronic, and advanced HF). Specifically, it evaluates MELD and MELD-XI scores, which integrate markers of both hepatic and renal dysfunction. Therefore, our study provides a more comprehensive and clinically relevant assessment of the prognostic significance of liver dysfunction in HF. This systematic review and meta-analysis aimed to assess the association between liver dysfunction markers, particularly MELD and MELD-XI scores, and mortality outcomes in patients with HF. By synthesizing data from observational cohort studies, this study seeks to clarify the prognostic significance of hepatic dysfunction in HF and to provide a more robust estimate of its impact on patient outcomes.

## Review

Materials and methods

A systematic search of electronic databases was conducted to identify studies examining the association between liver dysfunction markers and mortality in patients with HF. Electronic databases, including PubMed, Google Scholar, and the Cochrane Library, were searched from inception until February 2026. The search strategy incorporated combinations of keywords and MeSH related to HF and hepatic dysfunction. Search terms included combinations of “heart failure”, “hepatic dysfunction”, “liver dysfunction”, “MELD score”, “MELD-XI”, “hepatic congestion”, and “mortality”. Boolean operators such as AND and OR were used to refine the search strategy. Where applicable, filters for human studies and the English language were applied. Reference lists of included articles and relevant review papers were manually screened to identify additional potentially eligible studies. Only English-language peer-reviewed publications were included in this review. No formal protocol was registered; however, the methodology was defined a priori.

Eligibility Criteria

Observational studies, including prospective or retrospective cohort studies, that evaluated patients with HF, assessing liver dysfunction using established markers such as the MELD score, MELD-XI, or related biochemical markers of hepatic dysfunction, reporting mortality outcomes in patients with HF, and providing sufficient data to extract or calculate hazard ratios (HRs) with corresponding 95% confidence intervals (CIs) were included in this review. Studies were excluded if they were case reports, case series, conference abstracts without full text, reviews, editorials, or studies conducted in non-HF populations. Studies that did not report effect estimates or provided insufficient data for quantitative synthesis were also excluded (Table 1).

**Table 1:** Eligibility criteria for study inclusion HF: heart failure, HR: hazards ratio, MELD: Model for End-Stage Liver Disease, MELD-XI: MELD excluding international normalized ratio, CIs: confidence intervals

Category	Inclusion criteria	Exclusion criteria
Population	Adult patients (≥18 years) diagnosed with HF	Pediatric populations (<18 years) or studies not involving HF patients
Exposure/predictor	Liver dysfunction assessed using validated markers such as MELD or MELD-XI scores	Studies not evaluating liver dysfunction markers
Outcome	Reported mortality outcomes in HF patients	Studies not reporting mortality or relevant clinical outcomes
Effect measure	Studies reporting HRs with 95% CIs or sufficient data for calculation	Studies without extractable effect estimates
Study design	Observational studies, including prospective or retrospective cohort studies	Case reports, case series, reviews, editorials, conference abstracts, and animal studies
Language and publication type	English-language peer-reviewed full-text articles	Non-English publications or unpublished studies

Study Selection

Two reviewers (H.I.C. and M.A.) independently screened titles and abstracts retrieved from the database search to identify potentially relevant studies. Studies that appeared eligible underwent full-text review to determine final eligibility in accordance with the predefined inclusion and exclusion criteria. Discrepancies between reviewers were resolved through discussion and consensus. The study selection process followed the Preferred Reporting Items for Systematic Reviews and Meta-Analyses (PRISMA) guidelines [20], and a PRISMA flow diagram was constructed to illustrate the number of studies identified, screened, excluded, and included in the final analysis.

Data Extraction

Data extraction was conducted using a standardized data extraction sheet. The following information was collected from each study: first author, year of publication, country of study, study design, sample size, characteristics of the study population including mean age and proportion of male participants, type of HF population, liver dysfunction marker evaluated, exposure definitions or cut-off values, outcome measures, effect size estimates (HRs), corresponding 95% CIs, and follow-up duration. When multiple liver dysfunction markers were reported within a study, relevant effect estimates were extracted separately when appropriate. Extracted data were subsequently verified to ensure accuracy before inclusion in the meta-analysis. In cases of missing or incomplete data, attempts were made to extract relevant information from available text, tables, or supplementary materials. No additional contact with study authors was performed.

Risk of Bias Assessment

The methodological quality of the included studies was evaluated using the Newcastle-Ottawa Scale (NOS) for observational studies [21]. This tool assesses the risk of bias across three domains: selection of study participants, comparability of study groups, and assessment of outcomes. Each study was assigned a score ranging from 0 to 9 points based on these criteria. Scores of 7 or more points were considered to indicate good methodological quality, while lower scores indicated a moderate-to-high risk of bias.

Data Synthesis and Statistical Analysis

Statistical analyses were performed using Review Manager (RevMan) version 5.4 (The Cochrane Collaboration, Copenhagen, Denmark). The primary effect measure was HR, with corresponding 95% CIs estimated using the Wald method. Because adjusted HRs from observational studies were synthesized, the pooled estimates were generated using the generic inverse variance approach. Although the included studies adjusted for different sets of confounders, we prioritized the most fully adjusted effect estimates reported in each study to minimize confounding. This approach is consistent with standard practice in meta-analyses of observational studies, where pooling of maximally adjusted estimates is preferred despite variability in adjustment models. Before analysis, HRs were converted to their natural logarithmic form, and corresponding standard errors were derived from the reported CIs.

Given the anticipated variation in study populations, HF phenotypes, liver dysfunction markers, and follow-up periods, a random-effects model was used for the primary meta-analysis. Between-study heterogeneity was evaluated using the Chi-square test and quantified with the I² statistic, with higher I² values indicating greater inconsistency across studies. I² values were interpreted as follows: 0-25% (low heterogeneity), 26-50% (moderate heterogeneity), 51-75% (substantial heterogeneity), and >75% (considerable heterogeneity). A p-value <0.10 for the chi-square test was considered indicative of statistically significant heterogeneity. Additional subgroup analyses were conducted according to liver dysfunction marker type and HF phenotype to explore potential sources of heterogeneity. A leave-one-out sensitivity analysis was also performed to assess the stability of the pooled estimates. Publication bias was explored through visual inspection of funnel plot symmetry.

Results

**Figure 1:**
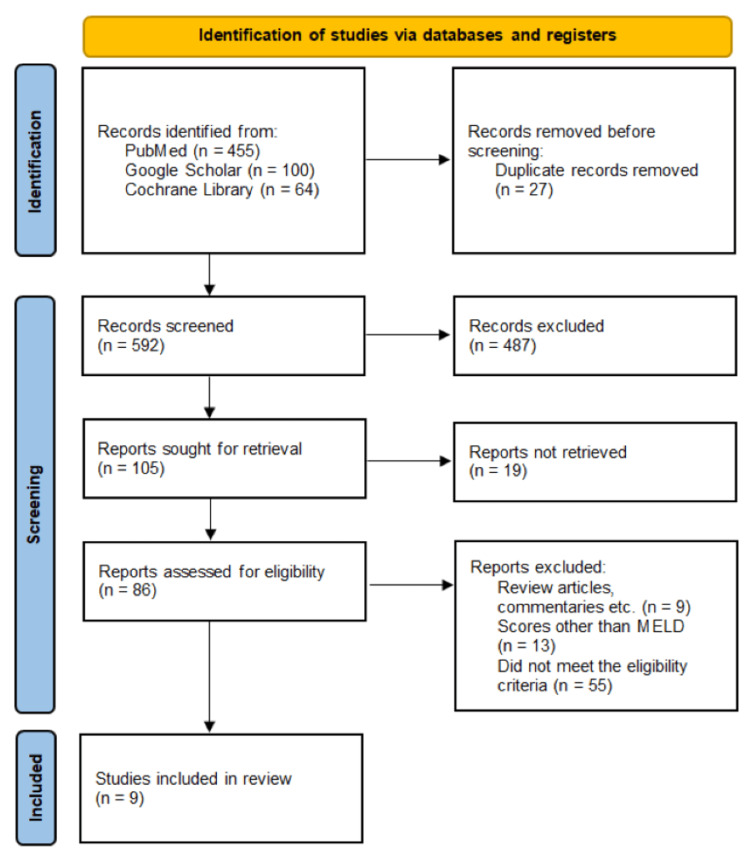
PRISMA flowchart PRISMA: Preferred Reporting Items for Systematic Reviews and Meta-Analyses, MELD: Model for End-Stage Liver Disease

A total of 619 records were retrieved from database searches in PubMed, Google Scholar, and the Cochrane Library. After removing 27 duplicate records, 592 studies remained for title and abstract screening. Of these, 487 studies were excluded as irrelevant to the research question. The full texts of 105 studies were assessed for eligibility. After applying the inclusion and exclusion criteria, nine studies were included in the final meta-analysis. The study selection process is summarized in the PRISMA flow diagram (Figure 1).

Study Characteristics

The nine included studies, published between 2013 and 2025, were conducted across several countries, including South Korea, Poland, Japan, China, and Italy. All included studies used observational cohort designs, including prospective and retrospective cohorts. The total number of participants across all studies was 33,316 patients with HF. The mean age of participants ranged from 56 to 79 years, and the proportion of male participants ranged from 41% to 78.9%. Several HF populations were represented, including chronic HF, acute HF, HFpEF, and advanced HF. Definitions of HF subtypes were not consistently reported across the included studies, and classification (e.g., acute vs chronic HF) was based on how these populations were described in the original articles. Only a limited number of studies provided explicit definitions. One study defined HFpEF according to the 2016 ESC Guidelines as left ventricular ejection fraction (LVEF) ≥50%. In another study involving advanced HF patients undergoing cardiac resynchronization therapy (CRT), response was defined by echocardiographic criteria, including an improvement in LVEF of ≥5% or a reduction in left ventricular end-systolic volume of ≥15% at 6 months. Additionally, one study defined advanced HF as a clinical syndrome characterized by persistent or progressive symptoms despite guideline-directed medical therapy, requiring advanced interventions such as cardiac transplantation, mechanical circulatory support, or palliative therapies. The remaining studies did not provide explicit definitions for HF subtypes. Liver dysfunction was assessed using validated scoring systems, primarily MELD and MELD-XI scores. Follow-up durations varied across studies, ranging from 60 days to three years (Table 2).

**Table 2:** Study characteristics of the included studies HF: heart failure, HFpEF: heart failure with preserved ejection fraction, LVEF: left ventricular ejection fraction, MELD: Model for End-Stage Liver Disease, MELD-XI: MELD excluding international normalized ratio, CRT: cardiac resynchronization therapy

Study	Country	Study design	Population	Sample size	Mean age	Male (%)	Liver dysfunction marker	Outcome	Follow-up
Kim et al., 2013 [17]	South Korea	Retrospective cohort	Ambulatory HF	343	56 ± 14	71%	MELD, MELD-XI	All-cause mortality	1 year
Biegus et al., 2016 [22]	Poland	Observational cohort	Acute HF	203	65 ± 12	76	MELD-XI	All-cause mortality	1 year
Matsue et al., 2019 [23]	Japan	Multicenter prospective cohort	Acute HF	1190	79 ± 12.5	41.40%	MELD-XI	1-year mortality	1 year
Kawahira et al., 2021 [14]	Japan	Prospective cohort	Acute HF	466	74 ± 13	58%	MELD-XI	Mortality	2.8 ± 1.5 years
Wang et al., 2021 [24]	China	Retrospective cohort	HFpEF	30,096	70.7 ± 12.8	64.10%	MELD-XI	Mortality	60 day in hospital
Lin et al., 2022 [25]	China	Retrospective cohort	Chronic HF	400	Median: 76 years	48.50%	MELD-XI	Mortality	3 years
Saito et al., 2022 [12]	Japan	Retrospective cohort	Advanced HF with reduced LVEF receiving CRT	283	67 ± 12	77.40%	MELD-XI	Mortality	Median 30 months
Curcio et al., 2025 [13]	Italy	Prospective cohort	Advanced HF	93	57.7 ± 11.6	82.80%	MELD	Mortality	31.4 ± 15.6 months
Jurkiewicz et al., 2025 [18]	Poland	Observational cohort	Decompensated HF	242	68 (66–74.6)	78.90%	MELD-XI	All-cause mortality	1.47 years

Overall, the studies demonstrated good methodological quality, with NOS scores ranging from 7 to 9. Matsue et al. [23] achieved the highest quality score of 9. Biegus et al. [22] and Jurkiewicz et al. [18] scored 8, indicating high methodological quality. The remaining studies each received NOS scores of 7. Overall, most studies demonstrated adequate participant selection, comparability of study groups, and reliable outcome assessment, suggesting a generally low risk of bias among the included studies.

Primary Analysis

**Figure 2:**
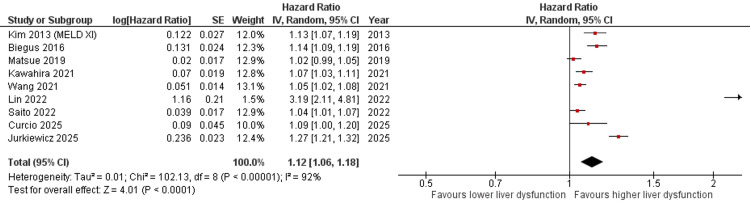
Forest plot illustrating the pooled HR for mortality associated with elevated liver dysfunction markers in patients with HF [12-14,17,18,22-25] HR: hazard ratio, HF: heart failure, CI: confidence interval, MELD-XI: Model for End-Stage Liver Disease excluding international normalized ratio

A meta-analysis of nine studies including patients with HF demonstrated that elevated liver dysfunction markers were significantly associated with increased mortality risk. The pooled HR was 1.12 (95% CI: 1.06-1.18, p < 0.0001) using a random-effects model. However, substantial heterogeneity was observed among the included studies (I² = 92%), likely due to differences in patient populations, liver dysfunction markers, and follow-up durations (Figure 2). Some included studies explicitly excluded patients with known primary liver disease, whereas others did not clearly report such exclusions, potentially contributing to heterogeneity and residual confounding.

Subgroup Analysis by Liver Dysfunction Marker

**Figure 3:**
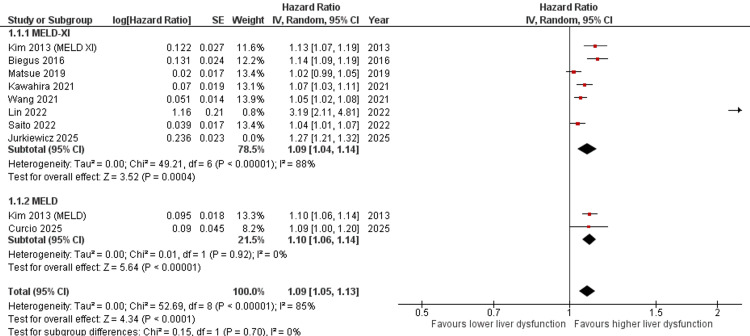
Subgroup analysis according to liver dysfunction marker type (MELD vs MELD-XI) and their association with mortality in HF patients [12-14,17,18,22-25] HF: heart failure, CI: confidence interval, MELD: Model for End-Stage Liver Disease, MELD-XI: MELD excluding international normalized ratio

Subgroup analysis based on liver dysfunction markers demonstrated that elevated MELD-XI scores were significantly associated with increased mortality risk in HF patients (HR = 1.09, 95% CI: 1.04-1.14, p = 0.0004). However, substantial heterogeneity was observed (I² = 88%). Studies evaluating MELD scores also showed a significant association with mortality (HR = 1.10, 95% CI: 1.06-1.14, p < 0.00001) with no heterogeneity (I² = 0%). The test for subgroup differences was not statistically significant (p = 0.70), indicating comparable prognostic performance between MELD and MELD-XI markers (Figure 3).

Subgroup Analysis by HF Type

**Figure 4:**
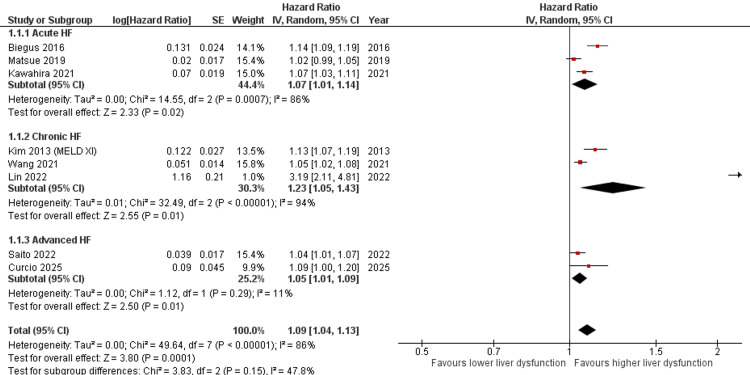
Forest plot showing subgroup analysis according to HF phenotype evaluating the association between liver dysfunction markers and mortality outcomes [12-14,17,22-25] HF: heart failure, CI: confidence interval, MELD-XI: Model for End-Stage Liver Disease excluding international normalized ratio

Subgroup analysis according to HF type demonstrated a significant association between liver dysfunction markers and adverse outcomes across all HF categories. In patients with acute HF, the pooled HR was 1.07 (95% CI 1.01-1.14; I² = 86%). For chronic HF, the pooled HR was 1.23 (95% CI 1.05-1.43; I² = 94%). In advanced HF, the pooled HR was 1.05 (95% CI 1.01-1.09; I² = 11%). Overall, the pooled HR across all studies was 1.09 (95% CI 1.04-1.13) with substantial heterogeneity (I² = 86%). The test for subgroup differences was not statistically significant (p = 0.15), suggesting no statistically significant difference in effect estimates across HF subtypes despite clinical heterogeneity (Figure 4).

Sensitivity Analysis

**Figure 5:**
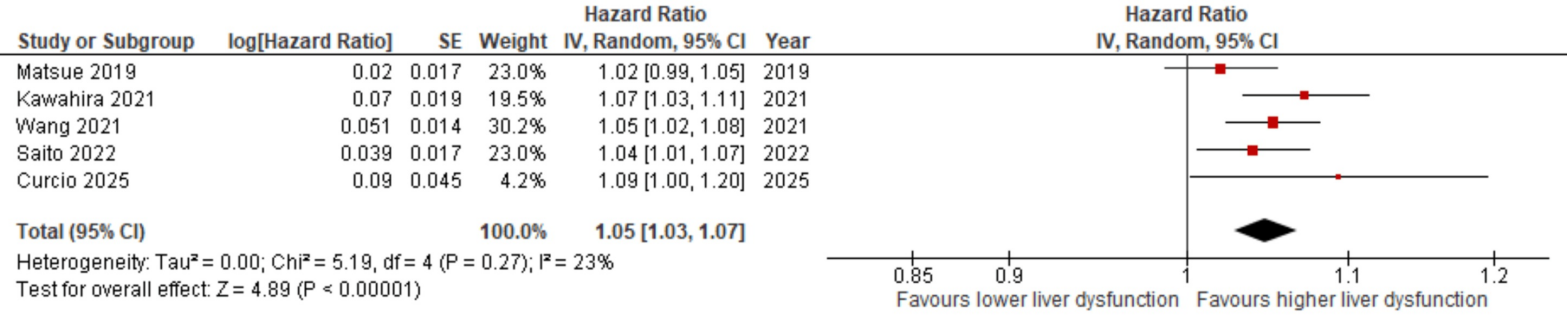
Sensitivity analysis evaluating the impact of sequentially excluding individual studies on the pooled HR for mortality related to liver dysfunction markers in HF [12-14,23,24] HR: hazard ratio, CI: confidence interval

Sensitivity analysis was performed to explore potential sources of heterogeneity. Sequential exclusion of four studies (Kim et al. [17]; Biegus et al. [22]; Lin et al. [25]; and Jurkiewicz et al. [18]) reduced heterogeneity substantially from I² = 92% to I² = 23%, while the pooled effect estimate remained statistically significant (HR = 1.05, 95% CI: 1.03-1.07). This suggests that the overall findings are robust despite variations across studies (Figure 5). These studies were identified as having the greatest influence on heterogeneity in the sensitivity analysis.

Publication Bias

**Figure 6:**
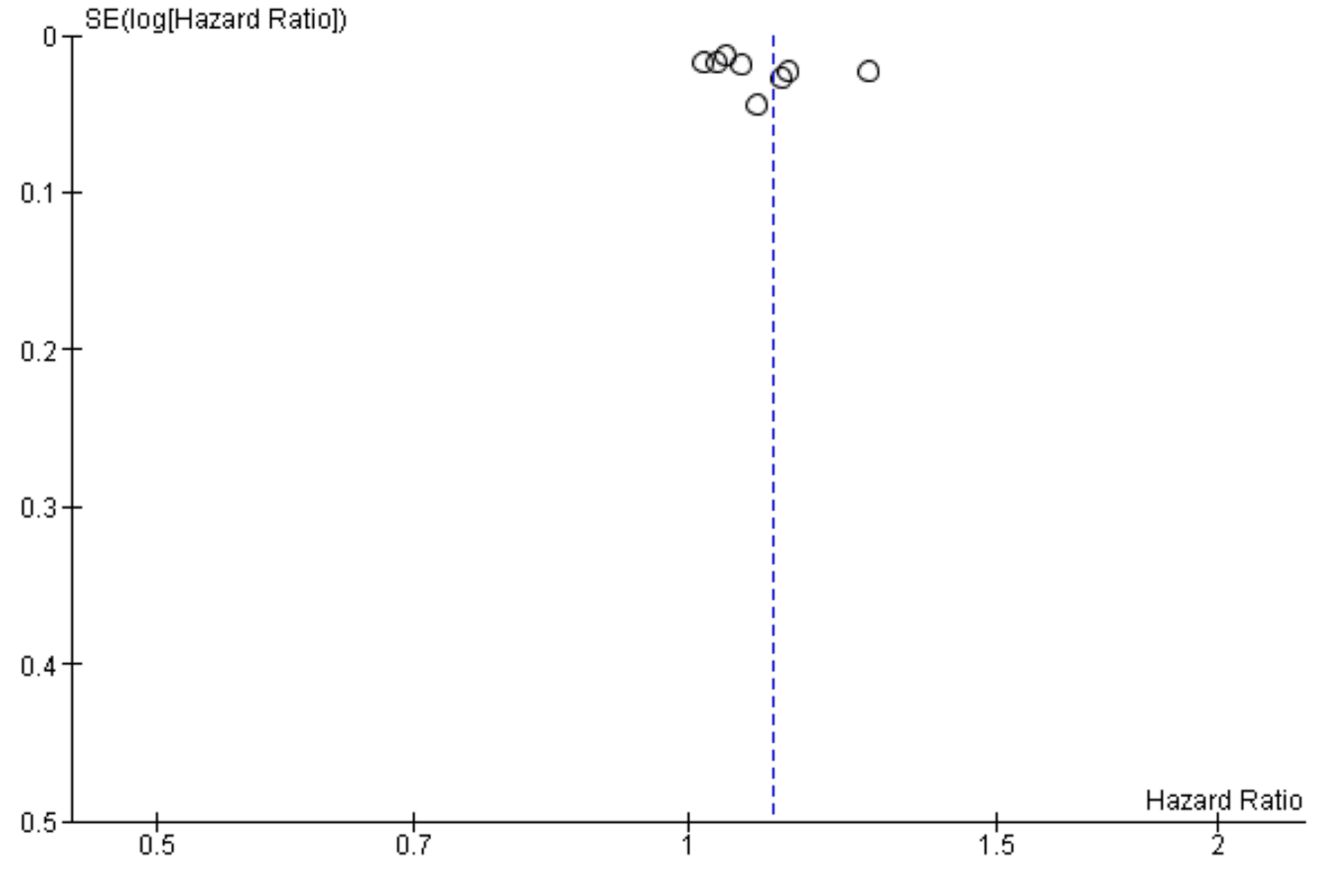
Funnel plot used to assess potential publication bias among studies investigating the relationship between liver dysfunction markers and mortality in HF HF: heart failure

Publication bias was evaluated through visual inspection of a funnel plot (Figure 6). The funnel plot demonstrated approximate symmetry, suggesting no clear evidence of small-study effects or publication bias. However, interpretation should be approached with caution due to the relatively small number of studies included in the meta-analysis.

Discussion

This meta-analysis demonstrates that liver dysfunction markers, including MELD and MELD-XI scores, are significantly associated with increased mortality in patients with HF. The association was consistent across different HF phenotypes, supporting the role of hepatic dysfunction as an important prognostic indicator in this population. Despite this, substantial heterogeneity was observed across studies, reflecting underlying clinical and methodological differences. Several pathophysiological mechanisms can explain the relationship between liver dysfunction and adverse outcomes in HFs. HF is a systemic disease that affects multiple organs, and the liver is particularly vulnerable to the hemodynamic disturbances associated with impaired cardiac function. Elevated right-sided heart pressures can lead to chronic hepatic congestion [26], while reduced cardiac output may impair hepatic perfusion and oxygen delivery [27]. These mechanisms contribute to hepatocellular injury, cholestasis, and progressive hepatic dysfunction [28]. Additionally, systemic inflammation and neurohormonal activation in HF may further exacerbate hepatic injury and contribute to multiorgan dysfunction [4]. As a result, abnormalities in liver function markers may reflect the severity of underlying cardiovascular impairment and may therefore serve as indicators of worse prognosis.

The findings of the present meta-analysis are consistent with several previously published studies reporting an association between hepatic dysfunction and poor outcomes in HF. Prior observational studies have demonstrated that elevated MELD or MELD-XI scores are associated with increased mortality, higher hospitalization rates, and worse overall prognosis in HF populations [29,30]. For example, studies evaluating MELD-XI scores in patients with advanced HF have shown that higher scores are predictive of adverse clinical outcomes and may help identify patients at increased risk of mortality [31]. Similarly, research in acute HF cohorts has suggested that liver dysfunction markers are independently associated with both short-term and long-term mortality [29]. By synthesizing evidence from multiple studies and diverse patient populations, the present meta-analysis provides stronger quantitative support for the prognostic value of hepatic dysfunction markers in HF.

Subgroup analysis based on HF phenotype demonstrated that the association between liver dysfunction markers and mortality remained significant across acute, chronic, and advanced HF populations, suggesting that hepatic dysfunction is a consistent prognostic indicator throughout the spectrum of HF. The slightly stronger association observed in chronic HF may reflect the cumulative effects of long-standing venous congestion and impaired hepatic perfusion, which contribute to progressive hepatic injury [4]. In patients with acute HF, hepatic dysfunction may occur due to transient hemodynamic disturbances and acute elevations in central venous pressure [32]. Meanwhile, in advanced HF, hepatic dysfunction may represent part of a broader pattern of multisystem organ impairment associated with severe cardiac disease [5]. Importantly, the test for subgroup differences was not statistically significant, indicating that the prognostic impact of liver dysfunction markers was broadly consistent across different HF stages. These findings support the concept of cardiohepatic syndrome, in which hepatic dysfunction develops as a consequence of chronic hemodynamic abnormalities in HF.

Our subgroup analysis comparing liver dysfunction markers further demonstrated that both MELD and MELD-XI scores were significantly associated with mortality outcomes. The MELD score was originally developed to predict survival in patients with end-stage liver disease and incorporates laboratory parameters such as bilirubin, creatinine, and the international normalized ratio (INR) [33]. However, many patients with HF receive anticoagulant therapy, which may influence INR values and limit the applicability of the traditional MELD score in this population. The MELD-XI score, which excludes INR, was therefore proposed as an alternative measure of hepatic dysfunction in cardiovascular populations [31,34]. The findings of this meta-analysis suggest that both scoring systems have prognostic value in HF. While MELD-XI may be more practical for patients receiving anticoagulation therapy because it excludes INR from its calculation, this observation is based on clinical rationale rather than direct evidence from the present subgroup analysis.

Despite the significant association observed in the pooled analysis, substantial heterogeneity was present across the included studies. Sensitivity analyses indicated that certain studies contributed more prominently to this variability, which may reflect differences in study populations, HF phenotypes, and methodological approaches. Variations in follow-up duration, baseline patient characteristics, and clinical management strategies may also influence the magnitude of the reported associations. Nevertheless, sensitivity analyses confirmed that the overall association between liver dysfunction markers and mortality remained statistically significant, suggesting that the findings of this meta-analysis are robust.

This study has several strengths. First, it provides a comprehensive synthesis of available evidence evaluating the prognostic significance of hepatic dysfunction markers in HF. By pooling data from multiple observational studies, the meta-analysis yields a large combined sample size, thereby improving statistical power and precision of the estimated effect size. Second, subgroup analyses were performed to explore potential sources of heterogeneity and to examine differences according to HF phenotype and liver dysfunction marker type. Third, sensitivity analyses were conducted to assess the stability of the pooled results.

However, several limitations should be acknowledged. All included studies were observational, which introduces the possibility of residual confounding and limits the ability to infer causality. A key limitation of this meta-analysis is that the included studies adjusted for different sets of confounding variables, which may affect the comparability of the pooled estimates. Although we used the most fully adjusted HRs from each study to minimize bias, residual confounding cannot be excluded. Substantial heterogeneity was observed in the primary analysis, likely reflecting differences in study populations, HF phenotypes, and follow-up durations. Additionally, variations in the cut-off values used to define elevated liver dysfunction markers may have contributed to variability in reported outcomes. The relatively small number of included studies also limited the ability to conduct more extensive subgroup analyses. Although visual inspection of the funnel plot did not suggest clear publication bias, the small number of studies restricts the reliability of this assessment.

## Conclusions

This review demonstrates that elevated liver dysfunction markers are significantly associated with increased mortality risk in patients with HF. The pooled analysis indicates that both MELD and MELD-XI scores serve as important prognostic indicators across different HF populations, including acute, chronic, and advanced HF. These findings support the concept of cardiohepatic interaction and highlight the potential value of liver dysfunction markers as tools for risk stratification in clinical practice. Although the association between hepatic dysfunction and adverse outcomes was consistent across studies, substantial heterogeneity was observed, reflecting variations in study populations, HF phenotypes, and follow-up durations. Nevertheless, sensitivity and subgroup analyses confirmed the robustness of the overall findings. Incorporating liver dysfunction markers into the clinical assessment of patients with HF may help identify individuals at higher risk of mortality and facilitate closer monitoring and more targeted management strategies. Future prospective studies are needed to further clarify the mechanisms underlying the relationship between hepatic dysfunction and HF outcomes and to determine how these markers can be integrated into existing prognostic models to improve patient care.
